# A Cross-Sectional Study on the Occurrence of the Intestinal Protist, *Dientamoeba fragilis*, in the Gut-Healthy Volunteers and Their Animals

**DOI:** 10.3390/ijms232315407

**Published:** 2022-12-06

**Authors:** Milan Jirků, Andrea Kašparová, Zuzana Lhotská, Miroslav Oborník, Kristýna Brožová, Klára J. Petrželková, Peter Samaš, Oldřiška Kadlecová, Christen Rune Stensvold, Kateřina Jirků

**Affiliations:** 1 Institute of Parasitology, Biology Centre, The Czech Academy of Sciences, Branišovská 31, 370 05 České Budějovice, Czech Republic; 2 Faculty of Science, University of South Bohemia, Branišovská 31, 370 05 České Budějovice, Czech Republic; 3 Institute of Vertebrate Biology, The Czech Academy of Sciences, Květná 8, 603 65 Brno, Czech Republic; 4 Department of Bacteria, Parasites and Fungi, Statens Serum Institut, 5 Artillerivej, 2300 Copenhagen, Denmark

**Keywords:** *Dientamoeba fragilis*, gut protist, survey, occurrence, Czech Republic, demography, human volunteers, animals, genotypes, sensitivity

## Abstract

*Dientamoeba fragilis* is a cosmopolitan intestinal protist colonizing the human gut with varying prevalence depending on the cohort studied and the diagnostic methods used. Its role in human health remains unclear mainly due to the very sporadic number of cross-sectional studies in gut-healthy populations. The main objective of this study was to expand knowledge of the epidemiology of *D. fragilis* in gut-healthy humans and their animals. A total of 296 stool samples from humans and 135 samples from 18 animal species were analyzed. Using qPCR, a prevalence of 24% was found in humans in contrast to conventional PCR (7%). In humans, several factors were found to influence the prevalence of *D. fragilis*. A more frequent occurrence of *D. fragilis* was associated with living in a village, traveling outside Europe and contact with farm animals. In addition, co-infection with *Blastocystis* spp. was observed in nearly half of the colonized humans. In animals, *D. fragilis* was detected in 13% of samples from eight species using qPCR. Our molecular phylogenies demonstrate a more frequent occurrence of Genotype 1 in gut-healthy humans and also revealed a likely a new protist species/lineage in rabbits related to *D. fragilis* and other related organisms.

## 1. Introduction

*Dientamoeba fragilis* is a human intestinal protist with cosmopolitan distribution and prevalence ranging from 0.2% to 82% (reviewed in [1,2,3]). Most studies addressing its occurrence are from high-income countries and surprisingly, less is known from low-income countries [1,2,4]. This protist was first described more than a century ago and was originally classified as a binucleate intestinal amoeba due to the absence of flagella (summarized in [5]). However, later morphological and molecular-phylogenetic analyzes revealed that *D. fragilis* is related to the flagellating trichomonads [2,5]. Nevertheless, some aspects of the life cycle of *D. fragilis* are still unclear. While colonization of the host hindgut by the trophozoite stage has been described in detail [2], the question of the mode of human-to-human transmission remains open. One hypothesis assumes involvement of the helminth vector with *Enterobius vermicularis* being the main candidate [6,7]. This is supported by observations such as (i) the colonization of a volunteer with *D. fragilis* after ingestion of *E. vermicularis* eggs derived from a donor colonized with both parasites [8], (ii) the molecular detection of *D. fragilis* DNA in *E. vermicularis* eggs [6,7], and (iii) also the frequent observation of co-infections of *D. fragilis* and *E. vermicularis* in children [9,10,11]. Another possibility of *D. fragilis* transmission has recently been discussed based on the description of a possible cyst stage [12,13], but this is considered controversial [14]. 

The role of *D. fragilis* in human health is currently debated in the medical and scientific community. In the past, this protist was neglected because of its apparent minor clinical significance [5], but some evidence of its possible pathogenic potential has accumulated over the last two decades [4]. Colonization with *D. fragilis* is often associated with gastrointestinal problems (e.g., [15,16,17]) and also with functional or inflammation-associated bowel diseases (Inflammatory Bowel Disease—IBD, Inflammatory Bowel Syndrome—IBS) [18,19,20,21]. That *D. fragilis* is a cause of some gastrointestinal diseases is often assumed due to the improvement of patients’ condition by its elimination after treatment [2]. However, these facts are not conclusive because, on the contrary, some studies reported that the elimination of *D. fragilis* had no effect on the condition of patients with gastrointestinal symptoms [22,23]. Unalan-Altintop et al. [24] stated that they found no association between the presence of *D. fragilis* and the development or exacerbation of ulcerative colitis or IBS. Another fact that may indicate a rather commensal role of *D. fragilis* is its frequent presence in the intestines of healthy individuals, based on the results of comparable or higher prevalence in healthy subjects in surveillance studies [25,26,27,28,29,30]. In addition, *D. fragilis*, along with another intestinal protist *Blastocystis* spp., is associated with higher diversity of the intestinal bacteriome, a marker of a healthy intestinal microbiome [31].

Host specificity and genetic diversity are among the other biological aspects of *D. fragilis* that remain to be elucidated. Regarding the host range, *D. fragilis* has only been detected in a few non-human species, most likely due to the lack of molecular epidemiological studies and the small number of animal species studied. To date, *D. fragilis* has only been detected in domestic pigs [32,33], non-human primates [34,35,36,37], cats and dogs [38], rats [39], and more recently in cattle [40] and pet budgerigars [41]. As part of the search for suitable experimental models for *D. fragilis*, laboratory mice also appear to be a susceptible host [12,42]. 

Genetic diversity of *D. fragilis* appears to be generally low. So far, only two genotypes have been distinguished, specifically Genotype 1 and Genotype 2 (reviewed in [1]). Moreover, currently available sources also show a low level of genetic polymorphism (i.e., about 3%) in the most studied *D. fragilis* genes to date, such as SSU rRNA, EF1α, and actin. This low genetic variability has been demonstrated for the above genes using phylogenetic approaches, analyzed both individually [1] and in concatenated form in the case of EF1α and actin [43]. Of the two known genotypes, Genotype 1 is the most common in the human population based on the available *D. fragilis* isolates from different regions around the globe [11,32,44]. In these surveys, Genotype 1 has been associated with both asymptomatic and symptomatic cases in individuals of different ages [4,11,45,46] and in patients suffering from IBS [24]. In contrast, Genotype 2 appears to be very rare in humans, resulting in limited data on this type [43,44]. This suggests that humans are probably not a natural host for Genotype 2 and the animal reservoir has not yet been discovered, due to the very limited number of studies on the occurrence of *D. fragilis* in animal hosts. To date, the possible zoonotic transmission of *D. fragilis* has been considered only between domestic pigs and their keepers originating from the same farm, based on molecular-sequence analyses of SSU rRNA and 5.8S rRNA gene fragments [32]. The sequences obtained showed high concordance with the defined Genotype 1 sequence from the GenBank database.

Since epidemiological surveys of *D. fragilis* in the gut-healthy human population are very rare, the main objective of the present study was to expand knowledge about the occurrence and prevalence of *D. fragilis* in such subjects in the Czech Republic, i.e., in a high-income country. We also investigated the presence of *D. fragilis* in animals with which these subjects were in close contact to evaluate potential zoonotic transmission. In humans, we then examined the association between the presence of *D. fragilis* and various factors such as lifestyle (traveling, living in urban versus rural areas), contact with animals, age, and gender. In addition, using the data from our previous study, Lhotská et al. [47], we were able to investigate the frequency of co-infection of *D. fragilis* and *Blastocystis* spp.

## 2. Results

We obtained a total 431 samples of which 296 samples were from humans and 135 samples were from animals (Table 1). Most of the non-human samples were from dogs (54/135), followed by cats (19/135), horses (15/135), rabbits (13/135), ruminants (12/135), chickens (8/135) and pigs (3/135), as well as some other animal species (11/135) (summary in Table 1).

Human samples were divided into eight consecutive age categories (see Table 2 for details); most samples were from individuals older than 18 years (244/296). We obtained 124 samples from men (42%; 124/296) and 172 from women (58%; 172/296) from 14 regions of the Czech Republic. All subjects confirmed that they do not suffer from any gastrointestinal symptoms or Inflammatory Bowel Disease (IBD). Regarding travel activity, 52 subjects reported not travelling outside the Czech Republic (18%; 52/296), whereas 140 subjects reported travelling within Europe (47%; 140/296), and 104 participants reported traveling outside Europe (35%; 104/296). Eighty-three were villagers (28%; 83/296) and the other 213 lived in town/city (72%; 213/296). In addition, 252 people (85 %; 252/296) reported recent contact with animals, of which 180 (71%; 180/252) had contact with pets only and 72 had contact with farm animals (29%; 72/252).

### 2.1. Prevalence of Dientamoeba fragilis Based on Two Molecular Approaches

Conventional PCR (cPCR) detected *D. fragilis* in 32 samples (31 from humans and one from animal) out of total 431 samples. However, Sanger sequencing confirmed its presence in only 23 samples, 22 of which were from humans and one from the animal host (rabbit). Only these verified samples were considered positive based on cPCR (Table 1). According to this, the overall prevalence was 7% (22/296) in humans and 0.7% in non-human hosts (1/135). We were unable to obtain sequences from the remaining nine samples, so they were classified as negative.

Real-time PCR (qPCR) detected *D. fragilis* in 72 human samples with a prevalence of 24% (72/296) and in 15 samples from animals (11%; 15/135). Positive animals included dogs (*Canis lupus familiaris*), cat (*Felis silvestris catus*), guinea pig (*Cavia aperea porcellus*), rabbits (*Oryctolagus cuniculus domesticus*), goat (*Capra aegagrus hircus*), sheep (*Ovis aries*), and horses (*Equus caballus*) (see Table 1 for details). All 23 positive samples detected by cPCR (confirmed by Sanger sequencing) were also positive in qPCR (see Table 3 for details). In addition, we verified the presence of *D. fragilis* in the qPCR amplicons of all qPCR-positive samples by Sanger sequencing. Because we did not have a culture of *D. fragilis* to generate a quantification curve in qPCR, only Ct values are shown for each positive sample (Table 3).

### 2.2. Comparison of the Sensitivity of cPCR and qPCR for Dientamoeba fragilis Detection 

We compared the sensitivity of these two methods on a set of human samples only (N = 296). The McNemar test showed that qPCR was a significantly more sensitive method for diagnostics of *D. fragilis* (*p* < 0.001; χ^2^ = 49; Supplementary Data S1). In addition, qPCR was also more successful in detecting *D. fragilis* in animal samples (Table 3), but these were not included in the statistical analyses.

### 2.3. Influence of the Specific Factors on the Occurrence of Dientamoeba fragilis

An effect of the specific factors on the distribution on *D. fragilis* was tested on the set of human samples only (N = 296) and statistically evaluated (for details see Section 4.7).

#### 2.3.1. Lifestyle

All 296 human samples were tested for the effect of lifestyle on *D. fragilis* colonization. Two factors were selected for this investigation: (i) urban life (town/city) versus village life, and (ii) impact of traveling (Table 4). For the latter factor, we distinguished three categories of individuals: (a) travelers outside Europe (104/296), (b) travelers only in Europe (104/296), and (c) non-travelers (52/296; Table 4). Regarding the living locality, a higher prevalence was observed in individuals living in village areas (29%, 24/83), compared to individuals from urban areas (23%, 48/213; Table 4), but this difference was not statistically significant. We observed a higher prevalence of *D. fragilis* in travelers. The prevalence among travelers within Europe was 24% (34/140), while 29% (30/104) was in travelers outside Europe (Table 4). However, the prevalence in non-travelers was significantly lower (15%, 8/52; *p* = 0.0324; Table 4).

#### 2.3.2. Contact with Animals

The overall prevalence of *D. fragilis* in the group of volunteers who reported contact with animals was 25% (62/252; Table 4). Among those without animal contact, the prevalence was 23% (10/44, Table 4). Based on the information from questionnaires, we divided the group of subjects with animal contact into (i) those with contact with pets (180/252) and (ii) those with contact with farm animals (72/252; Table 4). We found a significant difference in the incidence of *D. fragilis* between these two groups (*p* = 0.004; Table 4), specifically between 36% prevalence (26/72) in the group with contact with farm animals and 20% prevalence (36/180) in the group with contact with pets. 

#### 2.3.3. Age and Gender

We obtained samples in eight age categories, specifically 0–3, 4–6, 7–12, 13–17, 18–30, 31–49, 50–60, >60 years (Table 2). The highest prevalence of *D. fragilis* was observed in the 4–6 years group (50%, 7/14) and the lowest number of positive individuals was found in children under three years of age (11%, 2/18; Table 2). However, no significant differences were found between age categories (Fisher’s exact test; *p* = 0.91). The same conclusion was reached when the effect of age (0–79 years) was modelled as a continuous variable (Generalized linear model; χ^2^ = 0.30, *p* = 0.59). Pairwise comparisons of proportions between all age categories also revealed no significant differences using the binominal exact test with *p*-values adjusted by the Holm method (all *p* > 0.34; Table 2). Regarding gender effects there was no difference in the prevalence of *D. fragilis* between men (24%, 30/124) and women (24%, 41/172). 

### 2.4. Occurrence of Dientamoeba fragilis in the Family Environment

We obtained samples from several family members from a total of 69 families. Colonization of *D. fragilis* was detected in 34 families (i.e., 49%; Table 5). In addition, *D. fragilis* was detected in some families whose members were in close contact with a pet or farm animal that was positive for *D. fragilis* in qPCR (i.e., rabbits, cat, horses, dogs, and small ruminants; Table 5). 

### 2.5. Coinfection of Dientamoeba fragilis with Blastocystis spp. 

Within the human samples, we detected 33 cases of co-infection of *D. fragilis* with *Blastocystis* spp. (based on data published in [47]) out of 72 human samples positive for *D. fragilis* (46%; 33/72). Eleven of these subjects were men and 22 were women. All of these volunteers reported frequent contact with animals. In addition, ten subjects reported traveling within Europe (30%), 18 outside Europe (55%), and only five (15%) did not travel abroad at all. Twelve subjects lived in the village, 21 in the city. Most positive samples for both protists fell into the 31–49 age category (33%; 11/33). This was followed by the 18–30 age category (21%; 7/33). Among *Blastocystis* subtypes, the presence of *D. fragilis* was most frequently associated with ST1 (10/33), ST2 (6/33), ST3 (5/33) and ST4 (4/33) subtypes. In addition, some co-infections with ST5 (1/33), ST6 (3/33), and ST7 (1/33) subtypes were detected. The *Blastocystis* subtypes was not identified in the remaining three samples.

### 2.6. Genetic Diversity of Dientamoeba fragilis Based on Molecular Phylogenies

For the phylogenetic analyses, a total of twenty partial sequences of the SSU rDNA of *D. fragilis* were examined, specifically 19 sequences from colonized humans and one from a rabbit (B174). In addition, the *Trichomonas* sp. sequence (B131) derived from a chicken was included in the final dataset. This dataset included ten contigs (B9, B13, B126, B224, B238, B288, B329, B338, B174, B131), eight forward sequences (B113, B326, B328, B341, B371, B393, B413, B418), and three reverse sequences (B24, B82, B345). All our sequences were cloned to avoid unclear multiple signals or chimeras. The DNA sequences obtained in this study were deposited in the GenBank^TM^ under accession numbers OP375680—OP375701.

The final dataset for phylogenetic analyzes was generated to cover the diversity of *D. fragilis* from different hosts and all sequences obtained were compared with other *D. fragilis* sequences obtained from GenBank^TM^ (AB692771-73, AY730405, JQ677148-162, KU939320, MN91483, MW130447-8, MZ405082, OM250406, ON242172, U37461) and sequences from related organisms such as *Histomonas meleagridis* (EU647884-7, AJ920323), *Parahistomonas wenrichi* (EU647888,-89) and various trichomonads (AJ920324, AY247748/-50, AY886846, AY055800-3, JX943576, HQ149970, KM246611, KX353945-46, KX977495, U17509-10). This tree was rooted with B131 “*Trichomonas*” (OP375701) as an outgroup.

**Figure 1:**
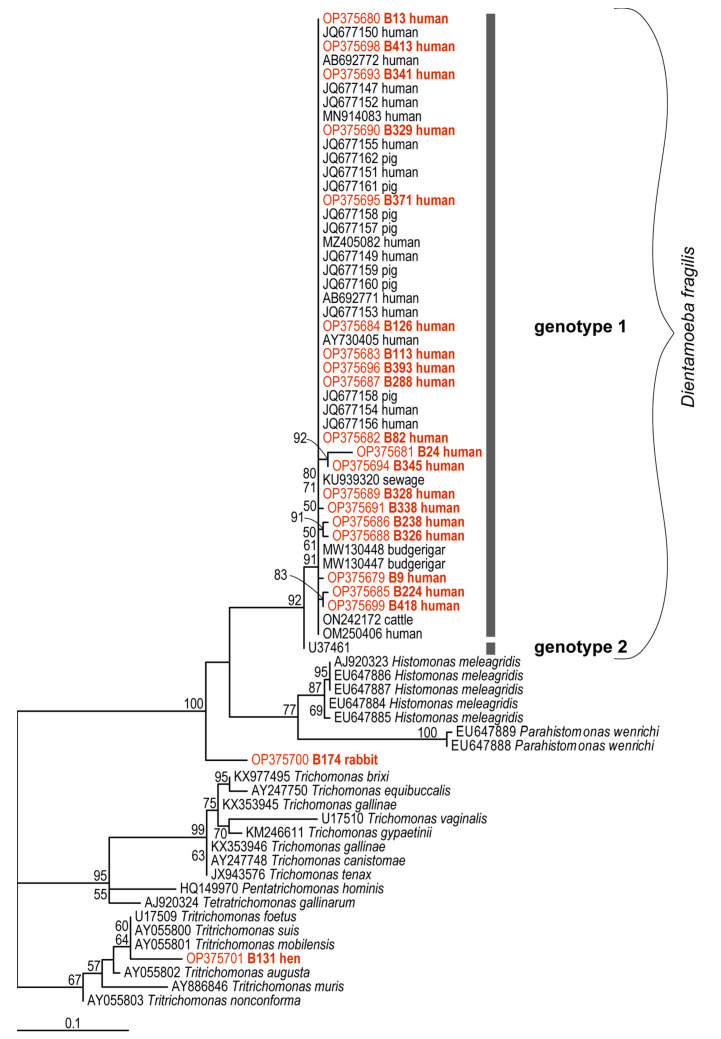
SSU rDNA tree from SSU rDNA sequences of *Dientamoeba fragilis* computed by IQTree. The numbers of the branches above represent maximum likelihood bootstrap supports as computed from 10,000 UFBoot. The scale bar represents 10 substitutions per 100 positions. New sequences are in red color.

The maximum likelihood tree (TVMe+G4+F, IQ-TREE) (Figure 1) shows a large clade containing *D. fragilis*-, *H. meleagridis*- and *P. wenrichi*-subclades, and then two trichomonad clades, one of which contains our sequence of *Trichomonas* sp. from chicken (B131/OP375701). The *D. fragilis* subclade includes our human sequences (OP375679-99), sequences defined as Genotype 1 from GenBank^TM^, and also contains only one Genotype 2 sequence (U37461) that is currently available in the GenBank database and it is represented by a separate branch (Figure 1). All our sequences from humans were unambiguously assigned to the Genotype 1 group, which has some internal polytomy (identical sequences) and three stable subgroups (one containing sequences OP375681, OP375694; the second containing sequences OP375686, OP375688; and the third with OP375685, OP375699). A very interesting result is the phylogenetic position of the sequence from rabbit (B174/OP375700). Although this sequence has a high identity with *D. fragilis* and *H. meleagridis* in the BlastNt database (concordance with *D. fragilis*: query coverage—70–76%, e-value—0.0, percent identity—89–92%; concordance with *H. meleagridis*: query coverage—75%, e-value—0.0, percent identity—89%), it forms a separate, strongly supported branch representing a new lineage of organisms closely related to *D. fragilis*, *H. meleagridis* and *P. wenrichi*. 

## 3. Discussion

To our knowledge, this is the first cross-sectional study addressing the occurrence of *Dientamoeba fragilis* in the Czech Republic in a set of stool and fecal samples obtained from gut-healthy volunteers and their animals of different species (see Table 1 for details). In humans, using real-time diagnostics (qPCR), we found an overall prevalence of 24% in a group of 296 humans. We also investigated the difference in sensitivity for detection of *D. fragilis* between qPCR and conventional PCR (cPCR) in human samples. Real-time PCR identified 50 more positive samples than cPCR, making it more sensitive for the detection of *D. fragilis*. The prevalence based on cPCR was 7%. Our observation is consistent with other studies comparing the sensitivity of molecular methods for the detection of other intestinal protists (e.g., [48]). In general, the diagnostics of *D. fragilis* are challenging and the sensitivity of different diagnostic methods can vary widely [49]. For example, Sharzhanov et al. [50] showed that the sensitivity of traditional microscopic methods for the detection of *D. fragilis* is lower compared to qPCR. Thus, qPCR appears to be the gold standard for *D. fragilis* diagnostics, and some recent studies already use this method routinely (reviewed in [1]).

Current data on the occurrence and prevalence of *D. fragilis* in asymptomatic individuals (i.e., subjects without gastrointestinal symptoms or disease) are generally very sporadic and come primarily from control groups of healthy subjects included in studies that focused on patients with various gastrointestinal problems (acute or chronic diarrhea, Inflammatory Bowel disease—IBD, or Inflammatory Bowel Syndrome—IBS). Because epidemiologic studies of *D. fragilis* use a variety of diagnostic approaches that may differ in specificity and sensitivity [1], we compared our data only with studies using qPCR. 

The available literature shows that the prevalence of *D. fragilis* in control groups of healthy individuals ranges from 36% to 71% [25,27,29,30,51,52]. For example, Heusinkveld et al. [52] conducted a large-scale cross-sectional survey to investigate the association between the occurrence of a number of enteropathogens (including selected protists) and acute gastroenteritis, and included more than 8500 pairs of a preschool-aged child and a parent living in the same household. They found that the prevalence of *D. fragilis* ranged from 36% to 39% in individuals without acute intestinal problems. Our results show a prevalence of 24% in gut-healthy people in different age groups, which is slightly lower than in the above-mentioned studies. Interestingly, a ten-year study conducted at the University Hospital of Parma demonstrated an almost comparable prevalence of *D. fragilis* (i.e., 20%) in patients with gastrointestinal symptoms [3]. Following data from patient cohorts and healthy subjects, the incidence of *D. fragilis* seems to be more or less comparable. Therefore, it is not possible to decide whether it is rather a pathogenic or a commensal organism. Determination of the so-called fecal protist load [48] could shed more light on deciphering the role of *D. fragilis* in human health. Such quantitative data could subsequently be compared between asymptomatic and symptomatic individuals, and in patients with IBD. Unfortunately, we were not able to quantify the fecal *Dientamoeba* load in our study because we did not have a monoxenic culture of *D. fragilis* to construct the quantification curve as was done in case of *Blastocystis* spp. in the study by Šloufová et al. [48].

It is important to mention that the prevalence of *D. fragilis* may be influenced by various factors, for example, age group selection, geographic location, or socioeconomic status [1,2]. The prevalence observed in our study may be affected by the wide age range in the group of volunteers because it ranged from 0 months to >60 years. 

Our set of human samples allowed us to observe the frequency of co-infections of *D. fragilis* with another intestinal protist *Blastocystis* spp. which was the subject of our previous study [47]. Based on the results of both studies, we found that almost half of the samples (i.e., 46%) from individuals colonized with *D. fragilis* had co-infection with *Blastocystis* spp. Interestingly, these cases of co-infections were associated with factors such as frequent contact with animals and traveling. Co-infection with these protists was also recorded in other studies that focused primarily on patients with gastrointestinal disease or symptoms, and the rate of co-infection rate varied widely. While several studies found co-infection rates ranging from 17.6% to 33.6% in patients with gastrointestinal symptoms [17,21,50,53], Yakoob et al. [20] detected co-infections in only 3% of IBS patients but found nothing comparable in a group of healthy subjects. In contrast, our results show a higher rate of co-infection in gut-healthy volunteers. Similarly, a higher co-infection rate of *D. fragilis* with *Blastocystis* spp. (i.e., 66.2%) was demonstrated in mostly asymptomatic children [54]. The authors of the latter study hypothesize that co-infection of *D. fragilis* and *Blastocystis* spp. is associated with healthy gut microbiome, as was the case in the study by de Boer et al. [26]. In addition, our data are unique because we were able to assess the presence *D. fragilis* and *Blastocystis* subtypes. *Dientamoeba* was most frequently detected in co-infection with ST1, ST2, ST3, and ST4 subtypes.

As far as we know, only one larger epidemiological study has been conducted for *D. fragilis*, covering a wider range of animal species [38]. However, *D. fragilis* was detected in only two fecal samples out of 420 samples and in two animal species (dog and cat) out of a total of 37 species. In our study, we found 13% of animals in close contact with human volunteers (135 samples obtained from 18 species) that were positive for *D. fragilis* using qPCR method. *Dientamoeba* was detected in eight animal species, several of which have already been described as potential hosts, specifically dogs (4/55), cat (1/19), and sheep (1/6). In dogs and cats, *D. fragilis* has been previously detected by PCR, whereas in sheep it was detected by traditional coproscopic methods [38,40,55]. In addition, we found *D. fragilis* in rabbits (5/13), horses (2/15), goat (1/4), and guinea pig (1/2). Surprisingly, we found no evidence of *D. fragilis* presence in domestic pigs, although a high prevalence (44–63%) has been recorded in the past [32,33]. 

Regarding the specific factors that might affect the distribution of *D. fragilis* in the gut-healthy human population, we examined the influence of living locality (urban versus rural), contact with animals, traveling (within and outside Europe, no traveling), age, and gender. Our results showed that prevalence was higher in people living in villages, and in travelers, especially outside Europe. Traveling is also considered an important predisposing factor for *D. fragilis* colonization in several other studies that focused on patients with diarrhea [15,16] or in a cross-sectional study in children under six years of age [29]. Interestingly, in a study by Norberg et al. [15], there was recorded a trend toward a higher incidence of *D. fragilis* in more than half of the patients with a history of traveling, and almost half of them had traveled outside Europe.

Not much is known about the effect of living locality; only Heusinkveld et al. [52] found a correlation between more frequent occurrence of *D. fragilis* and less urbanized areas. They also described a correlation between the occurrence of *D. fragilis* and the ownership of dogs. In contrast, Jokelainen et al. [29] concluded that contact with animals, especially those species considered potential hosts, had no effect on the spread of *D. fragilis*. Similarly, in our study, we found no significant effect of general contact with animals on the spread of *D. fragilis* in humans, but we showed a possible effect of keeping some farm animals, as more than one third of their breeders were colonized. Unfortunately, our data do not allow us to assess zoonotic transmission because we confirmed the presence of *D. fragilis* in animals only by qPCR without any subsequent sequence information (detailed information further). So far, the only case of possible zoonotic transmission was described in the study by Cacciò et al. [32], in which Genotype 1 was confirmed in pigs and their keepers based on molecular analyses of partial SSU- and 5.8S-rDNA sequences within one farm.

Age is generally assumed to have a significant impact on the distribution of *D. fragilis* in humans [1], but this has not always been confirmed [4,30]. In general, a higher prevalence is thought to occur in children up to about 15 years of age [29,51,56]. Although our results showed a more frequent occurrence of *D. fragilis* in the group of children younger than 12 years, these differences were not statistically significant, as in other studies [17,49,57]. Here, this trend could be due to an imbalanced number of samples collected from children under 18 years of age. The occurrence of *D. fragilis* in the other age groups was very similar. Regarding gender, we did not observe a difference in the incidence of *D. fragilis* between men and women, as was the case in the recent study by Sarzhanov et al. [50]. Nevertheless, findings of higher *D. fragilis* prevalence in women of childbearing age are frequently described [4,58].

A total of 69 families provided us with samples from all or several of their members so that we could track the occurrence of *D. fragilis* within the family. In almost half of the families (49%), the presence of *D. fragilis* was detected in at least one member, but usually in several members. Our results suggest that *D. fragilis* appears to circulate among family members or that positive family members may have been colonized from the same source. In six families, we also detected the presence of *D. fragilis* in their animals. However, based on our data, we were unable confirm zoonotic transmission.

The genetic diversity of *D. fragilis* appears to be low, and so far, only two genotypes have been proposed based on the variability of three genes (SSU rRNA, EF1, actin) [16,43,59,60]. However, most studies dealing with molecular or phylogenetic identification of *D. fragilis* isolates describe a predominant occurrence of Genotype 1 in different geographical areas, in different human cohorts (symptomatic versus asymptomatic individuals) and in animals [16,24,40,41]. Our nineteen SSU rDNA sequences of *D. fragilis* obtained from human samples also belong to Genotype 1 according to our phylogenetic analyses, and the sequences appeared in the phylogenetic tree together with Genotype 1 sequences from GenBank. Nevertheless, this Genotype 1 group exhibits some small genetic variability. In addition to the identical sequences forming a polytomy, we identified three highly supported subgroups. Analysis of the entire SSU rDNA sequence or another gene—as suggested by Simone M. Cacciò [1]—would perhaps provide more insight into the genetic diversity of *D. fragilis*.

In contrast to the human isolates, we were not able to obtain SSU rDNA sequences of *D. fragilis* by cPCR from most of the positive animals (with the exception of one sequence from a rabbit), apparently due to the low intensity of colonization, considering the Ct values obtained in qPCR (for details, see Table 3). Therefore, the presence of *D. fragilis* in these samples was confirmed only by qPCR followed by sequencing of very short qPCR amplicons (∼97 bp). Therefore, we were not able to determine whether it was indeed *D. fragilis*. To determine the genotypes of *D. fragilis* in animals, the partial SSU rDNA sequences obtained by cPCR (∼850 bp) are required. So far, only the occurrence of Genotype 1 has been described in pigs, cattle and budgerigars [40,41,44].

The fact that these 850 bp-SSU rDNA sequences are absolutely essential for the definitive genetic identification of *D. fragilis* in animals is also underlined by our finding of an apparently new protist in a rabbit, that appears to be closely related to *D. fragilis*. This SSU rDNA sequence was assigned to *D. fragilis* using the BlastNt database, but our phylogenetic analyzes indicated that it is the strongly supported separate branch next to *D. fragilis*, *Histomonas meleagridis*, and *Parahistomonas wenrichi* within one clade. These results suggest that it may be a new lineage or genus of a related protist that has not been described previously. However, further studies focusing on detailed morphological and phylogenetic-molecular identification is required for a taxonomic description. Based on all these facts, there is also a need to optimize or develop a new diagnostic qPCR protocol that is highly specific for *D. fragilis*.

## 4. Materials and Methods

### 4.1. Sample Collection and Ethical Approval 

The present study was conducted between 2017 and 2019 in the Czech Republic and follows the previous study on *Blastocystis* spp. [47] with identical sampling strategy and sample dataset. Briefly, stool samples were collected from healthy volunteers, none of whom had gastrointestinal symptoms or intestinal inflammatory diseases (inflammatory Bowel Disease-IBD, or Inflammatory Bowel Syndrome—IBS) at the time of sampling. To investigate possible zoonotic transmission, fecal samples were also collected from animals, with which the volunteers were in a close contact. All participants completed a questionnaire that included information on living locality (city/village), traveling (non-travel/travel in Europe/travel outside Europe), contact with animals (no contact/contact with pets only/contact with pets and livestock), gender and age (age categories: 0–3, 4–6, 7–12, 13–17, 18–30, 31–49, 50–60, >60) (for more details see [47]⁠). 

Each participant signed an informed consent declaration to participate in the study. The procedure, conditions, and ethical rules of this study are in accordance with the Declaration of Helsinki 2013 (World Medical Association). All data were strictly anonymized and processed in accordance with applicable laws of the Czech Republic (e.g., Act No 101/2000 Coll and subsequent regulations). The study was approved by the Ethics Committee of the Biology Centre of the Czech Academy of Sciences (reference number: 1/2017).

### 4.2. DNA Extraction and Samples Processing

Total DNA was extracted directly from stool/fecal samples using the commercial kit PSP Spin Stool DNA Kit (Stratec, Germany) according to the manufacturer’s protocol. All collected samples were subjected to two molecular diagnostic procedures such as conventional PCR (cPCR) [16] and real-time PCR (qPCR) [61]. As a positive control, we used DNA from the xenic culture of *D. fragilis*. This DNA was provided to us by our colleague Prof. J. Kulda from Charles University (Prague, Czech Republic).

### 4.3. Conventional PCR

Conventional PCR for detection of *D. fragilis* was performed using primers DF400 (5′–TATCGGAGGTGGTAATGACC–3′) and DF1250 (5′–CATCTTCCTCCTGCTTAGAC–3′) which amplify the ∼850 bp region of SSU rDNA [16]. PCR was performed in the T100TM Thermal Cycler (Biorad, Hercules, CA, USA) under the following conditions: 95 °C/5 min, 34 × (94 °C/1 min; 58 °C/1,5 min; 72 °C/2 min), and 72 °C/10 min. All PCR reactions were prepared in a final volume of 10 μL containing 5 μL of commercially produced 2× concentrated Master Mix (AccuPowerR Taq PCR PreMix; Bioneer, Daejeon, Republic of Korea), 1 μL of each primer, 1 μL of MiliQ water and 2 μL of template DNA. PCR products were visualized by electrophoresis, loading 8 μL of PCR product on a 1% agarose gel with ethidium bromide using the Thermo Fisher Scientific electrophoresis system (Waltham, MA, USA). 

### 4.4. Real-Time Diagnostics

Real-time PCR was performed using LightCycler LC 480 I (Roche, Basel, Switzerland) with a 96-well block under following conditions—95 °C/12 min; 50 × (95 °C/15 s; 60 °C/30 s; 72 °C/30 s) [61]⁠, using Master Mix (HOT FIREPol^®^ Probe qPCR Mix Plus Rox, Solis BioDyne, Tartu, Estonia). *Dientamoeba*-specific primers and probe set for part of SSU rRNA gene consisted of forward primer Df-124F (5′–CAACGGATGTCTTGGCTCTTTA–3′) and reverse primer Df-221R (5′–TGCATTCAAAGATCGAACTTATCAC–3′), amplifying the ∼97 bp region and Taqman probe Df-172 (FAM-5′–CAATTCTAGCCGCTTAT-3′-MGB) (Generi Biotech, Hradec Králové, Czech Republic). All negative samples were tested for PCR inhibition by addition of foreign DNA (obtained from experimental rat tissues) and a specific qPCR protocol (commercial primers and Taqman probe for detection of the rat gene for beta-2 microglobulin; Thermofisher Scientific). 

### 4.5. Cloning and Sequencing of Positive Samples

Sequences were always obtained from positive samples using cloning using pGEM^®^-T Easy Vector System I (Promega, Madison, WI, USA) followed by Sanger sequencing by a commercial company (Eurofins GATC Biotech, Konstanz, Germany). PCR amplicons of the appropriate size were purified using the GenElute^TM^ Gel Extraction Kit (Sigma-Aldrich, St. Louis, MO, USA) and sequenced in both directions using PCR primers. All sequences were then processed using the Geneious Prime 2019.0.4. software and subsequently compared with sequences in the GenBank^TM^ database (National Centre for Biotechnology Information) using BLASTn. 

### 4.6. Phylogenetic Analysis

The newly obtained SSU rRNA gene sequences were aligned to the available homologs from GenBank using the Clustal Omega algorithm in SeaView [62]. Alignment was manually edited in BioEdit [63] removing gaps, ambiguously aligned and hypervariable positions. Maximum likelihood trees were constructed using a TVMe+G4+F evolutionary model (selected by ModelFinder [64]) implemented in IQ-TREE [65]. Branch support was estimated from 10,000 UFBoot (ultrafast bootstrap approximation) [66] using the above software and conditions.

### 4.7. Statistical Analysis 

Difference in sensitivity of cPCR and qPCR for detection of *D. fragilis* was evaluated using the McNemar test with Yates’s correction. We employed Fisher’s Exact Test to examine differences between age categories in the incidence of *D. fragilis* in humans detected by qPCR. In addition, we used a generalized linear model to examine the effects of age as a continuous variable. The chi-square test was used to statistically evaluate the significance of the difference in *D. fragilis* prevalence between groups of humans within a single observed factor, such as lifestyle (urban versus rural), traveling (within and out of Europe), contact with animals (with and without), and gender. These analyzes were performed using GraphPad Prism 5.0 software and R Studio version 3.6.2. A *p*-value of less than 0.05 was considered to indicate statistical significance.

## 5. Conclusions

The prevalence of *D. fragilis* found in our study in the gut-healthy individuals suggests that the mere presence of this protist should not generally lead to efforts to eliminate it from the intestine, especially in asymptomatic individuals. This fact has been also considered for other intestinal protists, for example *Blastocystis* spp. [67]. Moreover, it is very difficult to decide whether *D. fragilis* is more of a commensal or a pathogen, since the frequency of its occurrence in patients with gastrointestinal symptoms is more or less comparable to healthy cohorts. It seems that the information about the presence of *D. fragilis* is not sufficient to assess its role in human health and that a quantitative indicator, the so-called fecal protist load, should be introduced into its diagnostics [48]. Such quantitative data on *D. fragilis* colonization and their comparison between different cohorts could facilitate understanding of its role in human health and disease. In addition, knowledge about *D. fragilis* in gut healthy individuals in high-income countries and in the human populations with traditional lifestyles needs to be expanded, including also knowledge about correlations with intestinal bacterial diversity. Worth mentioning is recent evidence of regulatory capabilities of some intestinal eukaryotes, including protists, which may be critical to the host intestinal ecosystem and immune system [68,69,70]. *Dientamoeba fragilis* may have a similar regulatory function, either alone or in co-infection with *Blastocystis* spp. but this remains to be tested experimentally. Finally, it is important to emphasize that to understand the zoonotic transmission of *D. fragilis*, it is necessary to monitor its occurrence in a range of animal species and also to decipher its genetic diversity.

## Figures and Tables

**Table 1:** List of human and animal species included in this study and the number of positive *Dientamoeba fragilis* samples for each species. (N—number of samples; cPCR—conventional PCR; qPCR—real-time PCR).

Host	Category	N	N Positive in cPCR	N Positive in qPCR
*Homo sapiens*	human	296	22	72
*Canis lupus familiaris*	dog	54	0	4
*Felis silvestris catus*	cat	19	0	1
*Equus caballus*	horse	15	0	2
*Oryctolagus cuniculus domesticus*	rabbit	13	1	5
*Ovis aries*	sheep	6	0	1
*Capra aegagrus hircus*	goat	4	0	1
*Cavia aperea porcellus* *Bos primigenius taurus*	guinea pig cow	2 2	0 0	1 0
*Sus scrofa domestica*	pig	3	0	0
*Gallus gallus domesticus*	poultry	8	0	0
*Testudo horsfieldii*	turtle	2	0	0
*Psittacus erithacus*	parrot	1	0	0
*Columba livia domestica*	pigeon	1	0	0
*Phodopus sungorus*	hamster	1	0	0
*Atelerix albiventris*	hedgehog	1	0	0
*Anas platyrhynchos domesticus*	duck	1	0	0
*Anser anser domesticus*	goose	1	0	0
*Phelsuma madagascariensis*	felsuma	1	0	0

**Table 2:** Descriptive summary of incidence and proportion of colonized individuals in all age categories detected by qPCR. (N—number of samples obtained; N positive—number of positive samples in qPCR; SD—standard deviation).

Age	N	N Positive	Incidence (%)	SD
0–3	18	2	11	0.323
4–6	14	7	50	0.519
7–12	16	7	44	0.512
13–17	4	1	25	0.500
18–30	73	15	21	0.407
31–49	88	21	24	0.429
50–60	36	9	25	0.439
60+	47	10	21	0.414

**Table 3:** Summary of qPCR-positive human and animal samples (N = 90) for *Dientamoeba fragilis* with Ct values and comparison with conventional PCR (cPCR) results.

Sample	Methods				Sample	Methods			
	cPCR	qPCR	Ct Value	Host		cPCR	qPCR	Ct Value	Host
B45	−	+	15	human	B437	−	+	34	human
B9	+	+	16	human	B286	−	+	35	human
B13	+	+	17	human	B289	−	+	35	human
B126	+	+	17	human	B372	−	+	35	human
B226	−	+	17	human	B375	−	+	35	human
B288	+	+	17	human	B49	−	+	36	human
B312	−	+	17	human	B34	−	+	37	human
B328	+	+	18	human	B378	−	+	37	human
B24	+	+	19	human	B246	−	+	37	human
B373	+	+	19	human	B31	−	+	38	human
B224	+	+	20	human	B150	−	+	38	human
B339	+	+	20	human	B287	−	+	38	human
B371	+	+	20	human	B41	−	+	39	human
B32	−	+	22	human	B121	−	+	39	human
B345	+	+	22	human	B227	−	+	39	human
B418	+	+	23	human	B234	−	+	39	human
B82	+	+	24	human	B245	−	+	39	human
B113	+	+	24	human	B291	−	+	39	human
B238	+	+	24	human	B325	−	+	39	human
B347	−	+	24	human	B327	−	+	39	human
B414	−	+	24	human	B331	−	+	39	human
B329	+	+	25	human	B416	−	+	39	human
B343	−	+	25	human	B3	−	+	40	human
B413	+	+	25	human	B23	−	+	40	human
B326	+	+	26	human	B35	−	+	40	human
B341	+	+	26	human	B42	−	+	40	human
B393	+	+	26	human	B106	−	+	40	human
B225	−	+	27	human	B232	−	+	40	human
B361	−	+	27	human	B174	−	+	23	rabbit
B338	+	+	28	human	B263	−	+	28	rabbit
B377	+	+	28	human	B262	−	+	29	guinea pig
B424	−	+	28	human	B151	−	+	31	rabbit
B346	−	+	29	human	B163	−	+	32	rabbit
B367	−	+	29	human	B422	−	+	33	goat
B124	−	+	30	human	B285	−	+	34	rabbit
B231	−	+	30	human	B229	−	+	38	cat
B338	−	+	31	human	B351	−	+	38	sheep
B374	−	+	31	human	B137	−	+	39	dog
B425	−	+	31	human	B152	−	+	39	rabbit
B430	−	+	31	human	B171	−	+	39	horse
B431	−	+	31	human	B337	−	+	39	dog
B406	−	+	32	human	B369	−	+	39	dog
B280	−	+	33	human	B205	−	+	40	horse
B419	−	+	33	human	B273	−	+	40	dog

**Table 4:** Prevalence of *Dientamoeba fragilis* in human samples according to the specific categories such as lifestyle (village versus city life, traveling) and contact with animals (pets, farm animals). (Samples N—number of samples obtained in each category out of the total number of samples). * Total number of volunteers in contact with animals. # Statistically significant differences, for details see Section 2.3.1 and Section 2.3.2.

Category	Samples N *	Prevalence	Category	Samples N *	Prevalence	Category	Samples N *	Prevalence
**living locality**						**non-travelers**		
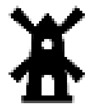 **village**	83/296	**29%**	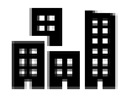 **city**	213/296	**23%**	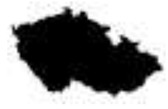 **only in the Czechia**	52/296	**15% #**
		(24/83)			(48/213)			(8/52)
**contact with animals**						**travelers**		
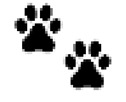 **in contact with animals**	252/296	**25%**	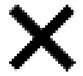 **no contact with animals**	44/296	**23%**	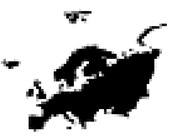 **inside Europe**	140/296	**24% #**
		(62/252)			(10/44)			(34/140)
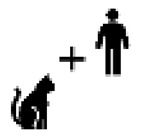 **in contact with pets**	180/252 *	**20% #**	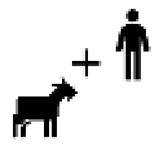 **in contact with farm animals**	72/252 *	**36% #**	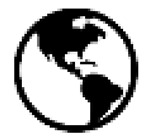 **outside Europe**	104/296	**29% #**
		(36/180)			(26/72)			(30/104)

**Table 5:** Overview of families including individuals colonized by *Dientamoeba fragilis* as well as *D. fragilis*-positive animals with which these families were in close or frequent contact (N family members—total number of family members; N positive members—number of family members colonized with *D. fragilis*; contact animal—animals colonized by *D. fragilis* which were in close contact with family members).

N Family Members	N Positive Members (Sample)	Contact Animal (Sample)
3	3 (B371, B372, B373)	dog (B369), horse (B337)
4	3 (B338, B339, B341)	sheep (B351)
3	2 (B418, B419)	goat (B422)
4	1 (B150)	rabbit (B151)
4	1 (B49)	rabbit (B285)
1	1 (B227)	cat (B229)

## Data Availability

Publicly available datasets were analyzed in this study. This data can be found in the GenBank^TM^ database under these accession numbers [OP375679-OP375701].

## Supplementary Materials

The following supporting information can be downloaded at: https://www.mdpi.com/article/10.3390/ijms232315407/s1.
