# Supplementary material for: A Cross-Sectional Study on the Occurrence of the Intestinal Protist, Dientamoeba fragilis, in the Gut-Healthy Volunteers and Their Animals
Source: Int J Mol Sci. 2022 Dec 6;23(23):15407. doi: 10.3390/ijms232315407 (PMC9737029; doi:10.3390/ijms232315407)
Supplement: Supplementary file 1 [file ijms-23-15407-s001.zip › ijms-1997711-supplementary.pdf]

Supplementary material

Supplementary Data S1. Comparison of the sensitivity of conventional PCR (cPCR) and real-time PCR (qPCR) for the detection of Dientamoeba fragilis in human samples (N=296) using McNemar test ($p < 0.05$; $\chi 2 = 49$).

		qPCR		
		positive	negative	
cPCR	positive	22	0	22(7 %)
	negative	50	224	274 (93 %)
		72 (24 %)	224 (82 %)	296
